# Review: Enhancing the Bioavailability and Stability of Anthocyanins for the Prevention and Treatment of Central Nervous System-Related Diseases

**DOI:** 10.3390/foods14142420

**Published:** 2025-07-09

**Authors:** Lan Zhang, Yan Wang, Yalong Cao, Fangxu Wang, Fang Li

**Affiliations:** School of Medicine, Jiangsu University, Zhenjiang 212013, China; 19805662010@163.com (L.Z.); 3232602031@stmail.us.edu.cn (Y.C.); 18997711949@163.com (F.W.)

**Keywords:** anthocyanins, bioavailability, stability, central nervous system

## Abstract

Central nervous system diseases are highly complex in terms of etiology and pathogenesis, making their treatment and interventions for them a major focus and challenge in neuroscience research. Anthocyanins, natural water-soluble pigments widely present in plants, belong to the class of flavonoid compounds. As natural antioxidants, anthocyanins have attracted extensive attention due to their significant functions in scavenging free radicals, antioxidation, anti-inflammation, and anti-apoptosis. The application of anthocyanins in the field of central nervous system injury, particularly in neurodegenerative diseases, neurotoxicity induced by chemical drugs, stress-related nerve damage, and cerebrovascular diseases, has achieved remarkable research outcomes. However, anthocyanins often exhibit low chemical stability, a short half-life, and relatively low bioavailability, which limit their clinical application. Recent studies have found that the stability and bioavailability of anthocyanins can be significantly improved through nanoencapsulation, acylation, and copigmentation, as well as the preparation of nanogels, nanoemulsions, and liposomes. These advancements offer the potential for the development of anthocyanins as a new type of neuroprotective agent. Future research will focus on the innovative design of nano-delivery systems and structural modification based on artificial intelligence. Such research is expected to break through the bottleneck of anthocyanin application and enable it to become a core component of next-generation intelligent neuroprotective agents.

## 1. Introduction

The central nervous system consists of the brain and spinal cord, which is the most important part of the nervous system. Central nervous system diseases include injury, infection, stroke, genetic defects, neurodegenerative diseases, and so on. They have many causes and unknown pathogenesis mechanisms, and their harm to patients is great while the prognosis of patients is poor. Since 1990, the number of nervous system disease cases has risen by 59%. In 2021, 3.4 billion people worldwide suffered from nervous system diseases, equivalent to 43.1% of the global population. This indicates that central nervous system diseases have a high prevalence rate worldwide, with global disability-adjusted life year (DALY) counts attributed to these conditions increasing by 18.2% between 1990 and 2021 [1]. The disease state, disability, and premature death caused by nervous system diseases have more a significant impact than cardiovascular diseases. They result in 443 million DALY, making them the leading cause of the global disease burden [1]. Moreover, the self-repair capacity of the central nervous system after injury is quite limited, and there are currently no effective treatment options. This issue requires global attention and intervention.

Anthocyanins, natural water-soluble pigments widely present in plants, belong to the class of flavonoid compounds [2]. They exhibit potent antioxidant, anti-inflammatory, anti-apoptotic, and anti-cancer activities, thereby safeguarding neurons from damage [3,4,5,6]. Numerous in vivo and in vitro studies have shown that anthocyanins can ameliorate several neurodegenerative diseases, such as Alzheimer’s disease (AD), Parkinson’s disease (PD), and amyotrophic lateral sclerosis (ALS) [7]. Extracts from foods rich in anthocyanins have been utilized in the development of dietary supplements. For instance, anthocyanins extracted from purple corn are employed as antioxidant dietary supplements, serving as excellent health promoters. However, due to the unique structural characteristics of anthocyanins, they are susceptible to various factors such as pH, temperature, light, metal ions, and microorganisms, which results in their poor stability [8,9,10,11,12]. Moreover, in in vivo studies, the bioavailability of anthocyanins has been estimated to be merely around 0.26–1.8% [13]. The bioavailability of anthocyanins in plasma was limited after the extraction of a substance rich in anthocyanins [14]. Therefore, the relatively low stability and bioavailability of anthocyanins are major obstacles that limit their widespread and effective application. Current research indicates that anthocyanins, as bioactive food components, exhibit correlations between their bioavailability and health maintenance as well as disease prevention capabilities. Recent studies have discovered that the stability and bioavailability of anthocyanins can be enhanced through techniques such as nanoencapsulation, the preparation of nanogels, nanoemulsions, and liposomes, acylation, and co-coloration. Then anthocyanins can be developed into new neuroprotective agents. Therefore, this review aims to provide a comprehensive approach based on the literature, discussing the structure, properties, and sources of anthocyanins, their bioactivities in preventing and treating central nervous system-related diseases, their stability and influencing factors, the bioavailability of anthocyanins, and strategies for enhancing their stability.

## 2. The Structure, Properties, and Sources of Anthocyanins

### 2.1. Structure of Anthocyanins

**Figure 1:**
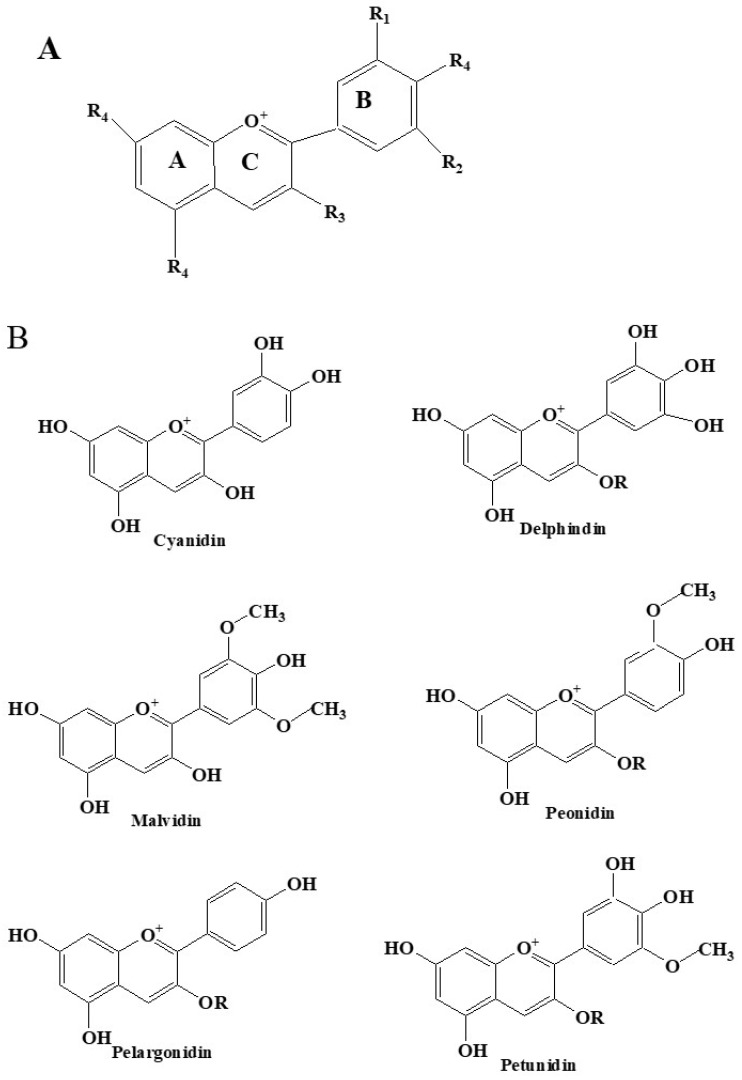
Common anthocyanin structures. (**A**) General flavonoid structure. In the structural formula of anthocyanins, R1 and R2 denote hydrogen, hydroxyl, or methoxy; R3 signifies hydrogen or a glycosyl group; and R4 represents hydroxyl or a glycosyl group. (**B**) Structures of the six most common anthocyanins. R represents glycosyl group. Common glycosyl groups include but are not limited to glucose, galactose, and rutinose.

The structure of anthocyanins, as illustrated in Figure 1, falls within the flavonoid class of compounds. Their fundamental carbon skeleton follows a C6−C3−C6 configuration, featuring two benzoyl rings (A and B) separated by an oxygen-containing six-membered heterocycle (C). This structure is characterized by a 3,5,7-trihydroxy-2-phenylbenzopyrylium cation molecular framework. Due to the variation in substituents at the R1 and R2 positions on the B ring (such as methoxy and hydroxyl groups), different types of anthocyanins can be formed [15]. Currently, there are twenty definite types of anthocyanins, six of which are particularly prevalent in plants: cyanidin, delphinidin, malvidin, peonidin, pelargonidin, and petunidin. Anthocyanins are highly reactive and inherently unstable, which is why they are rarely found in their free form in nature. They typically exist in a form where the free hydroxyl groups in their structure are linked to various monosaccharides and disaccharides via glycosidic bonds, forming diverse anthocyanins.

Anthocyanins possess unique chemical characteristics in their molecular structure, enabling them to exhibit a variety of biological activities, including antioxidant, anti-inflammatory, and neuroprotective properties. Their core structural advantages are reflected in the aspects outlined below.

The fundamental framework of anthocyanins is the 2-phenylbenzopyrylium ion (flavylium ion). Two phenyl rings (ring A and ring B) are connected through an oxygen-containing heterocyclic ring (ring C) to form a stable π-electron delocalization system, which endows them with strong antioxidant capacity (e.g., free radical scavenging and metal ion chelation). Ring A is typically substituted with 5,7-dihydroxyl groups (e.g., in cyanidin), while the number and position of hydroxyl groups on ring B (e.g., 3′,4′-dihydroxyl or monohydroxyl) determine their reduction potential and electron transfer efficiency, thereby influencing the strength of antioxidant activity [15].

### 2.2. Source of Anthocyanins

Anthocyanins are widely present in plants, and their concentrations exhibit significant variability influenced by factors such as plant variety, seasonal changes, climatic conditions, and developmental stage. The earliest and most abundant source of anthocyanins was the red pigment extracted from grape skins in red grape pomace. Subsequently, French scientist Masquelier discovered anthocyanins in the coating of peanut kernels. Nowadays, over 250 naturally occurring types of anthocyanins have been identified in various parts of plants, including stems, leaves, flowers, and fruits, across 27 families and 73 genera. Common sources of anthocyanins include grape, black wolfberry, eggplant, black rice, black bean, blood orange, figs, cherry, hawthorn, red cabbage, blueberry, purple sweet potato, strawberry, mulberry, red cabbage, flower tea, and other plant cell tissues [16,17,18,19,20,21,22,23]. These anthocyanins not only enrich the appearance of plants, but also play an important role in many biological processes.

### 2.3. Properties of Anthocyanins

There are many hydroxyl groups in the structure of anthocyanins, which are very polar. Therefore, anthocyanins have strong polarity and are easily soluble in water and more-polar solutions such as ethanol and methanol, and are not easily soluble in less-polar solutions such as diethyl ether and chloroform [8]. The color of an anthocyanin aqueous solution is greatly affected by pH, turning purple in a neutral environment, red in an acidic environment, and blue in an alkaline environment. Anthocyanins are sensitive to light and heat, can be destroyed by heating, and are stable in acidic environments [8,24]. Anthocyanins can form coordination complexes with cations such as Cu^2+^, Pb^2+^, Al^3+^, and Fe^3+^, altering their coplanar configuration, which subsequently modifies their color and stability [8,25]. The absorption peak of anthocyanins in the visible region is about 500 nm, while the absorption peak in the ultraviolet region is between 270 and 280 nm. These absorption peaks correspond to the conjugated structure of the anthocyanin molecule, which enables it to display characteristic colors at distinct wavelengths [26].

## 3. Anthocyanins Play a Role in the Prevention and Management of Central Nervous System-Related Diseases

**Figure 2:**
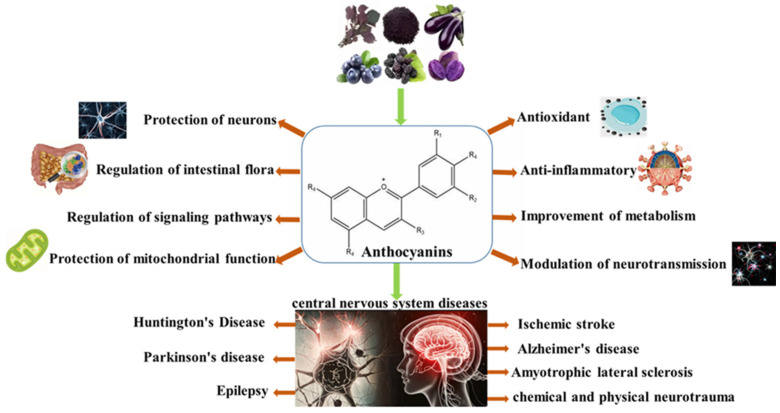
Anthocyanins play a role in the prevention and management of central nervous system-related diseases.

The complexity and intricacy of the central nervous system make it one of the most vulnerable systems in the body. Its health condition is directly related to an individual’s cognitive function, behavioral performance, and overall well-being. The unique structural features of anthocyanins—such as their polyhydroxylated conjugated system, pH-dependent isomerization, and diverse functional groups—allow these compounds to engage in redox reactions, metal ion binding, and targeted signaling pathway interactions. These properties underpin their promising role in combating central nervous system diseases. Studies have shown that anthocyanins have a variety of biological activities such as antioxidation, anti-inflammatory effects, and the regulation of glucose and lipid metabolism, which have an important impact on human health [27,28,29,30,31]. It is particularly noteworthy that anthocyanins can cross the BBB, exerting a direct protective effect on the central nervous system of the brain [32]. And their potential to improve cognitive function by modulating cholinergic neurotransmission has been shown, restoring the activities of sodium, potassium adenosine triphosphatase, and calcium adenosine triphosphatase, promoting neuronal regeneration, and preventing memory impairment [33,34,35]. This property makes anthocyanins show great application prospects in the prevention and treatment of central nervous system diseases (Figure 2).

### 3.1. Parkinson’s Disease

PD is an adult-onset progressive neurodegenerative disease characterized by clinical manifestations such as bradykinesia, resting tremor, rigidity, and postural instability. These symptoms will worsen as the disease advances [36]. These behavioral impairments not only affect the physical and mental health of middle-aged people and the elderly, but also impose a significant burden on both patients and society. PD has a complex pathogenesis, including oxidative stress, mitochondrial dysfunction, and the progressive degeneration of dopaminergic neurons in the substantia nigra pars compacta, leading to a decline in striatal dopamine levels and the aggregation of α-synuclein into Lewy bodies [37,38]. Genetic factors and environmental exposures also contribute to the pathogenesis of PD. Industrial or agricultural toxins with molecular structures similar to 1-methyl-4-phenyl-1, 2, 3, 6-tetrahydropyridine (MPTP), such as certain pesticides, herbicides, and rotenone, may be among the causative agents of PD [39]. These factors interact synergistically, leading to impairments in motor control. One study investigated the toxic effects of MPTP on the substantia nigra in squirrel monkeys after systemic administration. The results indicated that MPTP could selectively damage the dopaminergic neurons in the substantia nigra of squirrel monkeys, leading to the degeneration and death of these neurons [40]. PD is commonly chemically modeled using MPTP, which has been shown to induce the death of nigrostriatal dopaminergic neurons [41]. In experiments using mice as a model, it has been found that repeated low-dose exposure to paraquat can increase the production of reactive oxygen species in the mouse brain, activate microglial cells, and thereby lead to the degeneration of dopaminergic neurons in the substantia nigra. Paraquat can induce the tyrosine phosphorylation of parkin in the dopaminergic SH-SY5Y cell line and inhibit the Wnt/Wingless signaling pathway [42]. Epidemiological studies have shown that regular consumption of anthocyanin-rich foods, such as berries, can reduce the risk of developing PD [43]. Anthocyanins demonstrate potential in the prevention and treatment of PD through multiple mechanisms, including antioxidant and anti-inflammatory effects, neuroprotection, and the modulation of relevant signaling pathways. Pretreatment with a 70% ethanol extract of mulberry fruit (administered at 500 mg/kg per d for 15 d) prior to MPTP injection significantly reduced pro-apoptotic protein expression and prevented MPTP-induced dopaminergic neuron death, ultimately ameliorating Parkinsonian symptoms in mice [44]. In addition, recent studies in the same model further reported that treatment with Mulberry fruit lyophilized powder (administered by oral gavage, for 5 consecutive weeks) also significantly attenuated MPTP-induced increases in the expression of α-synuclein and ubiquitin (the main components of Lewy bodies) [45]. Strathearn et al. investigated whether extracts rich in anthocyanins, proanthocyanidins, or other polyphenols suppress the neurotoxic effects of rotenone in a primary cell culture model of PD. Dopaminergic cell death elicited by rotenone was suppressed by extracts prepared from blueberries, grape seed, hibiscus, blackcurrant, and Chinese mulberry. Extracts rich in anthocyanins and proanthocyanidins exhibited greater neuroprotective activity than extracts rich in other polyphenols, and a number of individual anthocyanins interfered with rotenone neurotoxicity. The concentration of all tested extracts was 10–50 μg/mL. The blueberry and grape seed extracts rescued rotenone-induced defects in mitochondrial respiration in a dopaminergic cell line, and a purple basal extract attenuated nitrite release from microglial cells stimulated by lipopolysaccharide. These findings suggest that anthocyanin- and proanthocyanidin-rich botanical extracts may alleviate neurodegeneration in PD via the enhancement of mitochondrial function [46]. Zaim et al. utilized MPP^+^ (1-methyl-4-phenylpyridinium ion) to induce cytotoxicity in human neuroblastoma SH-SY5Y cells, establishing an in vitro model of PD-associated neuronal toxicity. They demonstrated that black carrot anthocyanins exert cytoprotective effects by enhancing metabolic activity and reducing membrane damage at higher concentrations (50 μg/mL and 100 μg/mL). Furthermore, these anthocyanins protect dopaminergic neurons from reactive oxygen species (ROS)-mediated cell death through ROS scavenging, thereby improving PD [47].

### 3.2. Alzheimer’s Disease

AD is the most common type of dementia and is a chronic central neurodegenerative disease characterized by memory loss, cognitive dysfunction, and personality disorders. In a randomized controlled trial, anthocyanins significantly improved cognitive function in individuals at high risk for AD. These cognitive improvements showed significant correlation with reductions in serum IL-6 and CRP levels, suggesting that anthocyanins may delay cognitive decline through anti-inflammatory mechanisms, with particularly pronounced effects in individuals exhibiting elevated baseline inflammation levels [48]. Krikorian et al. found that daily supplementation with blueberry equivalents of 500–750 g (containing 300–500 mg anthocyanins) for 12–16 weeks significantly improves episodic memory and working memory performance in older adults with mild cognitive impairment. The underlying mechanisms involve multi-target pathways including oxidative stress regulation, neuroinflammation suppression, and the functional remodeling of brain networks [49]. Experimental studies using a rat model of memory impairment induced by intraperitoneal scopolamine injection (1–2 mg/kg) demonstrated that daily anthocyanin supplementation (50–200 mg/kg) significantly ameliorates memory deficits. The neuroprotective effects involve multi-target synergistic mechanisms, including the modulation of cholinergic system function, suppression of oxidative stress, and enhancement of synaptic plasticity [50]. A controlled intervention study utilizing the APPswe/PSEN1dE9 (APP/PS1) transgenic mouse model of Alzheimer’s disease demonstrated that chronic administration (16 weeks) of anthocyanin-enriched bilberry (*Vaccinium myrtillus*) and blackcurrant (*Ribes nigrum*) extracts (200 mg/kg/day, dietary supplementation) significantly reduced Aβ accumulation, and both extracts prevented cognitive decline and improved behavioral abnormalities [51]. Korean black bean anthocyanins (12 mg/kg/day, i.p. for 30 days) have also demonstrated beneficial effects in APP/PS1 transgenic mice, characterized by the activation of the phosphoinositide 3-kinase/protein kinase B (PI3K/Akt) signaling pathway and upregulation of nuclear factor erythroid 2-related factor 2 (Nrf2) antioxidant signaling. These mechanisms enhance neuronal survival and synaptic function, thereby improving cognitive performance [52]. Another study on a similar mouse model expressing a mutant Swedish variant of APPswe showed that an anthocyanin-rich pomegranate extract (a 4% pomegranate-supplemented diet for 15 consecutive months) reduced the levels of Aβ, interleukin-1β (IL-1β), and interleukin-6 (IL-6) in the brain of transgenic mice, in addition to the levels of proinflammatory cytokines such as tumor necrosis factor-alpha (TNF-α) [53]. Recent reports similarly demonstrated that anthocyanins or anthocyanin-3-O-glucosides significantly ameliorated cognitive deficits induced by amyloid-β injection and improved multiple measures of oxidative stress and neuroinflammation in rats and mice [54,55]. Moreover, treatment with 0.18% and 0.9% anthocyanin-rich extracts from mulberries for 12 consecutive weeks was found to preserve cognitive function and significantly reduce the amyloid plaque burden and stress kinase signaling in the brains of senescence-accelerated mice [56].

An anthocyanin-rich extract from grape skins (AC-12-R-WS-P/10120/Gin: 601412, 200 mg/kg, once daily, by gavage for 25 days) has also been shown to have a positive effect on a model of streptozotocin-induced dementia, which has recently been used as a model for sporadic AD. Anthocyanins were shown to enhance the antioxidant state within the brain and decrease the activity of ache, leading to improved behavioral performance in cognitive tests [57].

Several in vitro studies have also shown that anthocyanins and their extracts can enhance cell viability, reduce ROS and intracellular calcium levels, upregulate β-secretase expression, promote survival proteins, downregulate the elevation of pro-apoptotic signaling proteins, and significantly reduce inflammatory markers, such as nuclear factor kappa-light-chain-enhancer of activated B cells (NF-κB), inducible nitric oxide synthase, cyclooxygenase-2, TNF-α expression, and c-Jun N-terminal kinase activation, weakening Aβ toxicity [41,55]. Acai berry extract can reverse the cytotoxicity induced by Aβ25–35, including enhancing neuronal activity and reducing lactate dehydrogenase and reactive oxygen species levels, and improving the symptoms of AD by promoting neuronal autophagy to reduce neuronal damage [58].

### 3.3. Chemical and Physical Neurotrauma

With the advancement of societal development and the progression of medical care, certain chemical pharmaceuticals or physical stimuli have surpassed their intended scope of application, thereby subjecting individuals to prolonged exposure to environments characterized by chronic chemical or toxic stimulation. Long-term chronic stimulation can cause certain forms of damage to the nerve and even cause irreversible neurological dysfunction. Electromagnetic radiation can be natural or anthropogenic. It includes short, high-frequency waves (e.g., X-rays, gamma rays, and other forms of ionizing radiation) and long, low-frequency waves (e.g., ultraviolet, visible light, infrared, microwaves, television waves, radio frequency fields, and other non-ionizing radiation). The carcinogenic, leukemia-causing, and genetic effects on health of ionizing radiation are well known. Exposure to very-low-frequency electromagnetic fields induced alterations in glutamate levels, the activation of the Mitogen-Activated Protein kinase (MAPK) signaling pathway, and a reduction in cyclic Adenosine Monophosphate response element-binding Protein (CREB) phosphorylation in the hippocampal region of mice, ultimately leading to cognitive dysfunction. Radioactive substances are widely used in energy production, scientific research, medicine, industry, and other fields, and their serious hazards have attracted more and more attention. In recent years, due to the considerable side effects of antiradiation drugs, research on radiation-protective agents has gradually expanded from past chemical substances to the use of natural antiradiation drugs and functional foods. Mice were orally administered anthocyanins from lingonberry (200, 100, 50 mg/kg) once daily for 14 days before receiving 6 Gy gamma rays and being sacrificed on day 7 after irradiation. The results showed that pretreatment with anthocyanins from lingonberry significantly increased the thymus and spleen indexes and the survival rate of spleen cells, and significantly reduced the number of micronuclei in polychromatic red blood cells of bone marrow, indicating that anthocyanins from lingonberry had the potential for immune stimulation and could be resistant to immunosuppression induced by radiation [59].

### 3.4. Ischemic Stroke

Ischemic stroke is a cerebrovascular disease in which brain tissue is subjected to ischemia, hypoxia, and infarction due to the occlusion of blood vessels in the brain [60]. Stroke symptoms include sudden unilateral weakness, numbness, diplopia, slurry speech, ataxia, and nonorthostatic vertigo. Strokes can be sudden or progressive. The mechanism involves the disruption of blood flow leading to hypoxia of brain tissue, triggering glutamate excitotoxicity, oxidative stress, mitochondrial dysfunction, and cell death [61,62,63,64]. Epidemiologic investigations have shown that acute (2 h) and chronic (1 month) intake of freeze-dried whole cranberry powder equivalent to 100 g of fresh cranberries can improve endothelial function in healthy adults [65]. In a recent study, transient ischemic stroke was induced for 2 h by ligating the common carotid artery, external carotid artery, and internal carotid artery on the proximal middle cerebral artery. After the release of the arteries and reperfusion, a purple potato extract, which contained 209.8 milligrams of anthocyanin per 100 g, was administered to Wistar rats for 7 days. In the treatment group, brain-derived neurotrophic factor levels were increased, while apoptosis-inducing factor was significantly decreased. In addition, animals in this group had better neurological scores than those in the control group [66].

Other positive effects of anthocyanin preconditioning in inducing stroke have also been mentioned, such as improving spontaneous activity and memory, and reducing the levels of molecules involved in the inflammatory response such as TNF-a, IL-1B, and IL-6 [67,68]. Pure primary cultured cortical neurons from Sprague Dawley (SD) rats were exposed to oxygen–glucose deprivation and glutamate to mimic in vitro ischemia-induced cell death. Black soybean seed coat extract (up to 100 g/mL) was found to prevent membrane damage and inhibit ROS overproduction in a dose-dependent manner. Thus, the mitochondrial membrane potential of primary neurons exposed to oxygen–glucose deprivation is preserved. Astrocytes, a type of glial cell, serve as a reservoir of antioxidants and release essential neurotrophins, helping to maintain an optimal environment for neuronal function in the central nervous system and having a regulatory effect on neuropathological events. In mouse models, the loss of astrocyte function or altered viability leads to neuronal degeneration and destruction, which correlates with inflammation and infarct volume after stroke. Manolescu et al. analyzed the survival of Uppsala 87 Malignant Glioma human glioblastoma cells treated with black soybean anthocyanins under oxygen–glucose deprivation (mimicking ischemic conditions in the body) and found that anthocyanins increased cell viability in a dose-dependent manner, and that the increase in cell viability was associated with a decrease in ROS levels and protected cells by the activation of the autophagosome marker Microtubule-associated protein 1A/1B-light chain 3 [68].

### 3.5. Amyotrophic Lateral Sclerosis (ALS)

ALS is a neurodegenerative disease that primarily affects motor neurons, leading to progressive muscle weakness and atrophy. Symptoms include muscle weakness, muscle atrophy, fasciculation, bulbar palsy, and pyramidal tract signs. Multiple factors are involved in pathogenesis, including genetic mutations, chemical imbalances (e.g., glutamate excess), dysregulated immune responses, and abnormal protein processing [69,70]. The effect of oral administration of 2 mg/kg anthocyanin-rich strawberry extract for 55 consecutive days on disease onset and progression was evaluated using a G93A mutant human superoxide dismutase 1 (hSOD1G93A) ALS mouse model. Strawberry extract-supplemented hSOD1G93A mice had significantly delayed disease onset (about 17 days) and survived significantly longer (about 11 days) than untreated mutant mice. Furthermore, strawberry extract-treated hSOD1G93A mice showed increased grip strength throughout disease progression. Histopathological analysis showed that strawberry extract supplementation significantly reduced astrogliosis in the spinal cord and preserved the neuromuscular junction in the gastrocnemius muscle. These data suggest that anthocyanins have great potential as therapeutic agents in preclinical models of ALS [71].

### 3.6. Huntington’s Disease (HD)

HD is an autosomal dominant neurodegenerative disease. Symptoms include involuntary choreiform movements, muscle stiffness or contractures, abnormal eye movements, difficulty walking and remaining balanced, and difficulty speaking and swallowing. Cognitive symptoms involve decreased organization, attention, and ability to control impulses [72]. HD’s pathogenesis involves the production of mutant huntingtin protein, which leads to abnormal transcriptional regulation, mitochondrial dysfunction, and abnormal intracellular protein processing [72,73]. Dietary supplementation with anthocyanins delayed the onset of motor dysfunction in female R6/1 Huntington’s disease transgenic mice, and the levels of cholesterol oxidation products were also lower in the cortex of mice fed dietary supplementation with anthocyanins compared with those fed standard diets [74]. The anthocyanins were extracted from European blackcurrant (Ribes nigrum) berries, purified by HPLC to achieve >95% purity of two major monomeric components—cyanidin-3-glucoside (C3G) and delphinidin-3-glucoside—and administered via drinking water at a dose of 500 mg/kg/day. Anthocyanin treatment was initiated at 8 weeks of age and maintained until the endpoint (32 weeks).

Recent studies suggest that anthocyanins may interact with epigenetic regulatory elements, such as histone deacetylases (HDACs) and DNA methyltransferases (DNMTs). This interaction could open a new dimension of neuroprotection and provide a direction for the development of epigenetically antioxidant synergistic therapies. For example, anthocyanins can alter DNMT activity, leading to changes in DNA methylation patterns [75]. By influencing DNA methylation, anthocyanins may regulate the expression of genes related to the pathogenesis of AD, such as those involved in amyloid processing, inflammation, and neuronal function [76]. In a transient middle cerebral artery occlusion model in male mice, various HDAC inhibitors have been shown to reduce the extent of neuronal injury and improve functional outcomes [77]. A dose of 300 mg/kg of Valproic Acid administered subcutaneously 3 h after ischemia can reduce infarct volume by 32.7%. Additionally, a dose of 300 mg/kg of Sodium Butyrate administered subcutaneously 6 h after ischemia can also significantly reduce infarct volume. Sodium 4-Phenylbutyrate administered intraperitoneally 1 h after ischemia, at doses of 40 mg/kg and 120 mg/kg, can significantly reduce infarct volume. This indicates that modulating histone modifications through the inhibition of HDAC activity could be a promising therapeutic strategy for stroke. Anthocyanins, by influencing HDAC activity, may contribute to neuroprotection in stroke and other neurodegenerative diseases [78].

## 4. Instability of Anthocyanins and Its Influencing Factors

### 4.1. Structure of Anthocyanins

Anthocyanins belong to the class of flavonoids. The basic structure of this class of flavonoids is composed of benzopyranic cation parent nuclei, and its unique molecular structure directly determines its variable physical and chemical properties. Anthocyanins contain multiple phenolic hydroxyl groups and glycosidic bonds, and these active groups make them prone to oxidation, hydrolysis, and other chemical reactions. The parent core unit of anthocyanin lacks an electron, and its stability is mainly affected by the B-ring substituent in the structure. The presence of electron donor groups such as hydroxyl or methyl groups would reduce the stability of anthocyanins, with the least stability specifically under neutral conditions, and otherwise enable better stability [79]. It is particularly noteworthy that the presence or absence of a C2 hydroxyl group significantly affects stability—for example, geranionin (C2 without hydroxyl group) is more stable than cyanidin (C2 with hydroxyl group), which reveals a delicate balance between the number of hydroxyl groups and steric resistance in the molecular structure.

### 4.2. pH Value

The color and activity of anthocyanins are regulated by the environmental pH value. In an acidic environment (pH < 3), anthocyanins mainly exist as red flavylium cations; in neutral to weakly acidic environments (3 < pH < 8), the color of anthocyanins gradually changes from red to purple. This is due to the deprotonation of flavylium cations, which results in the formation of purple neutral quinone bases and blue anionic quinone bases, and the formation of colorless alcohol pseudobases. Under alkaline conditions (pH > 11), the quinone base of anthocyanin was transformed into the unstable divalent anion form with a blue-green color. When the pH value was higher than 11, anthocyanins appeared blue. At pH values above 12, anthocyanins were present mainly as yellow chalcones [80]. The differences in electronic distribution among these forms affect their interactions with biomolecules (e.g., DNA, enzymes) [14]. Under acidic conditions, the flavylium cation form of anthocyanins can bind to DNA through electrostatic interactions to form stable complexes. For example, cyanidin-3-O-glucoside can bind to DNA at pH 3, inhibiting the activity of DNA polymerase and thereby affecting DNA replication. Under neutral-to-alkaline conditions, the interaction of anthocyanins in the quinoidal base or chalcone structure with DNA is weaker. For example, cyanidin mainly exists in the quinoidal base form at pH 7, with weaker binding to DNA and less impact on its structure. In conclusion, the color of anthocyanins varies with pH, and the order of color change from acidic to basic is roughly red, purple, green, blue, and yellow. This color change allows anthocyanins to act as a natural acid–base indicator.

### 4.3. Temperature

Temperature is another factor that affects the stability of anthocyanin molecular structures. Heat treatment can cause anthocyanins to undergo various chemical changes, such as glycosylation, nucleophilic attack by water, cleavage, and polymerization. As a result, an increase in temperature leads to the loss of anthocyanins and the formation of degradation products [81,82,83]. On the other hand, high temperatures hydrolyze the pyrene ring of anthocyanins to produce chalkiness, which is the reason why anthocyanins turn brown under heat. Many studies have proved that the thermal degradation of anthocyanins conforms to first-order reaction kinetics, the color intensity and amount of anthocyanins decrease with time/temperature, while the amount of brown pigment/polymer fraction increases, and the dissociation rate of anthocyanins accelerates with the increase in temperature or the extension of the thermal degradation time [84,85]. For example, Turker et al. studied the effect of temperature on the stability of shalgam anthocyanins stored at 4, 25, and 40 °C for 90 days. When the temperature was the same, the monomer anthocyanin content and color intensity decreased with time, while the polymer color and percentage of browning increased. Acylated anthocyanins are more stable than nonacylated anthocyanins at all storage temperatures, with the highest retention of anthocyanins observed at 4 °C, with a half-life between 231 and 239 days [86]. Qu et al. observed a similar phenomenon: the antioxidant capacity of a pomegranate peel extract under sterilization treatment and storage at 4 °C retained 58% of its original scavenging activity over a storage period of up to 180 days [87].

### 4.4. Light and Oxygen

Light can also affect the stability of anthocyanins, and the degradation of anthocyanins in an aqueous solution will be accelerated when exposed to light [85]. The light-induced degradation of anthocyanin is mediated by a hydroxyl group at C-4 to produce an intermediate, which is then uncycled at C-2 to form chalcone. Chalcone is further degraded to benzoic acid and trihydroxybenzaldehyde, such as 2, 4, 6-trihydroxybenzaldehyde and 3,4,5-trihydroxybenzoic acid [88]. Ochoa et al. showed that the degradation of anthocyanin by light also conformed to the first-order kinetic reaction, and the degradation of anthocyanin was significantly different under light and dark conditions. The retention rate of anthocyanin was decreased and the degradation rate was highest when the anthocyanin was stored under outdoor light. The retention rate of anthocyanins under indoor light or dark storage was higher than that under outdoor light storage, and the degradation rate was lowest under dark storage [89]. In addition, anthocyanins are rapidly degraded by oxidative mechanisms in an oxygen environment [84]. It has been reported that red cabbage has a high degradation rate of anthocyanins under aerobic conditions. This degradation process involves complex oxidation reactions, which eventually lead to structural destruction and a reduction in anthocyanin molecular weight, which has an important impact on the stability and persistence of anthocyanins in nature [90].

### 4.5. Metal Ions

Different metal ions have different effects on the stability of anthocyanins. The addition of some metal ions could effectively improve the stability of anthocyanins. Some metal ions can also chelate with anthocyanins to form chelates and affect the biological activity of anthocyanins. Metal cations are able to alter the absorption spectrum of pyrrole rings by affecting the distribution of unlocalized electrons. The strongest color effects were observed when co-coloring was performed using positively charged alkaline earth metals or inactive metals (+2, +3) [84]. Among anthocyanins, only compounds from cyanidin, delphinidin, and morvianidin are able to undergo metal chelation due to the free hydroxyl group in the B ring. The most common metals capable of forming complexes with anthocyanins are Cu, Fe, Mg, Sn, and K [84,91,92]. In a neutral solution, Fe^2+^ ions have a dual effect on red cabbage anthocyanins. First, they stabilize the blue quinone-like structure of acylated anthocyanins by forming stable complexes, preventing their addition with water molecules and the autoxidation process. Second, Fe^2+^ ions that leak from weaker complexes formed with nonacylated and monoacylated anthocyanins promote auto-oxidation and lead to the rapid degradation of these pigments [93]. In contrast, the addition of other metal ions, including Sn^2+^, Fe^3+^, Al^3+^, and Ca^2+^, reduced the color and thermal stability of red cabbage anthocyanins. Among them, Sn^2+^ caused the highest degree of degradation of anthocyanins, while Al^3+^ had the least effect [94]. Similarly, red cabbage anthocyanin extract iron chelates were found to reduce the storage and thermal stability of anthocyanins [95].

The accumulation of metal ions is closely related to various neurodegenerative diseases and brain injuries. In Alzheimer’s disease, the accumulation of iron, copper, and zinc ions catalyzes the generation of free radicals, leading to oxidative stress and neuronal damage. In Parkinson’s disease, the accumulation of iron ions in the substantia nigra of the brain impairs mitochondrial function and affects dopamine metabolism. In amyotrophic lateral sclerosis, the accumulation of copper and zinc ions leads to mutations and the aggregation of superoxide dismutase 1, which damages motor neurons. During cerebral hemorrhage and cerebral infarction, the accumulation of iron and copper ions exacerbates brain damage. After traumatic brain injury, the accumulation of iron and zinc ions leads to oxidative stress and neuronal damage. The accumulation of these metal ions not only exacerbates oxidative stress but also affects the function of the nervous system. The polyhydroxyl structure of anthocyanins can selectively bind metal ions (such as Fe^3+^ and Cu^2+^). Chelation sites: The catechol group (3′,4′-dihydroxyl on ring B) and the carbonyl group on ring C form stable five- or six-membered chelation rings, which inhibit metal ion-mediated oxidative stress (e.g., the Fenton reaction). This plays a key role in the prevention and treatment of neurodegenerative diseases [96].

### 4.6. Enzymatic Reaction

There are two main types of anthocyaninase degradation: one is caused by endogenous degradation enzymes, which are present in plant tissues, and the other is caused by enzyme preparations used during processing or extraction [97]. Enzymes that normally cause anthocyanin degradation include β-glucosidase, peroxidase, and polyphenol oxidase [98]. By hydrolyzing the glycosidic bonds in anthocyanins, β-glucosidases form unstable anthocyanin intermediates, which are then rapidly converted to chalcone by a ring-opening reaction and further decomposed into phenolic compounds or subjected to further oxidation by polyphenol oxidase and peroxidase, resulting in color attenuation. Therefore, β-glucosidase is seen as a prerequisite for anthocyaninase degradation [99,100]. Polyphenol oxidase or peroxidase catalyzes the oxidation of o-diphenols to o-quinones, which further react to produce brown polymers. Polyphenol oxidases can directly oxidize the phenolic hydroxyl group in anthocyanins to generate the corresponding quinones. This oxidation reaction can cause the color of anthocyanins to gradually become dark and cause them to eventually lose their bright color. Polyphenols can also oxidize other phenolic compounds to generate quinones, which can co-oxidize anthocyanins and further accelerate the degradation of anthocyanins [91]. A coupled oxidation mechanism may be involved in the degradation of anthocyanins by peroxidases. For example, peroxidases in litchi peel could not directly oxidize anthocyanins in vitro, but the content of anthocyanins decreased rapidly after the addition of a guaiacol solution, indicating that peroxidases degraded anthocyanins through a coupling reaction with anthocyanins, phenolic compounds, and H_2_O_2_. The effect of peroxidase on anthocyanins is different from that on anthocyanins. Anthocyanins are more susceptible to peroxidase degradation, whereas anthocyanins are relatively stable due to the presence of glycosidic bonds [101].

### 4.7. Drying Process

In the drying process, the anthocyanin content will gradually decrease with the increase in drying temperature and time. Different drying methods had different effects on the retention of anthocyanins. For example, the highest retention of anthocyanins was observed under freeze-dried conditions, which was not significantly different from fresh fruit, while the lowest retention of anthocyanins was observed under hot-air-drying conditions [102]. Vacuum drying could retain anthocyanins better than hot-air drying. Under vacuum-drying conditions, anthocyanins were less exposed to oxygen due to the decrease in oxygen partial pressure, and the retention rate of anthocyanins was higher than that under hot-air drying, indicating that drying in a low-oxygen environment could improve the stability of anthocyanins. For example, during the hot-air and vacuum drying of mulberry fruits, hot-air drying at 60 °C and 75 °C resulted in the degradation of anthocyanins. The degradation of monomeric anthocyanins follows second-order kinetics. Thermodynamic parameters, including activation energy, enthalpy change, and entropy change, showed significant differences between hot-air drying and vacuum drying. The degradation of monomeric anthocyanins by vacuum drying showed higher kinetic rate constants and higher retention values [103]. In addition, it was found that microwave drying significantly improved the retention rate of anthocyanins by shortening the drying time, achieving uniform heating, reducing the oxidation environment, and protecting the structure of anthocyanins [104]. High hydrostatic pressure, as a non-thermal processing technology, applies high pressure (usually 100–1000 MPa) to food under normal or low-temperature conditions to achieve the purposes of sterilization and preservation of and improvement in food characteristics. This technique uses a liquid medium, such as water or oil, to transfer pressure so that the food is evenly stressed in all directions, thus avoiding the destruction of the nutritional composition and sensory quality of the food by traditional heat treatment [105]. Engmann et al. studied the effect of high hydrostatic pressure at different pressures (200 MPa, 400 MPa, 600 MPa) on the stability of anthocyanins in mulberry juice and showed that the retention of anthocyanin content was better at lower pressure levels than at higher pressure levels. The 200 MPa/10 min treatment retained 96.50% of anthocyanins [106].

### 4.8. Concentration Techniques

Dincer et al. compared the effects of thermal evaporation and osmotic distillation on the concentration of black mulberry juice, and found that the loss rate of anthocyanin in osmotic distillation and thermal evaporation was 6.5% and 16.2%, respectively, which indicated that osmotic distillation, as a mild concentration method, had little effect on the stability of anthocyanin [107]. Fazaeli et al. compared the effects of a rotary evaporator and vacuum microwave evaporator on the concentration of black mulberry juice, and found that under the same vacuum conditions, microwave heating could greatly shorten the concentration time, reduce the loss rate of anthocyanin, and better retain the color of black mulberry juice [108]. This indicates that microwave-assisted concentration technology can be used as an effective concentration method to reduce the loss of anthocyanins during the concentration process. In conclusion, mild concentration techniques such as osmotic distillation and microwave-assisted concentration help to reduce the loss of anthocyanins during the concentration process, thereby improving their stability.

## 5. Bioavailability of Anthocyanins

The biological efficacy of active compounds depends on different factors, including the food substrate, digestibility, bioavailability, solubility, transporters, molecular structures, and metabolic enzymes [109]. The bioavailability of a nutrient is the fraction of the effective concentration of the nutrient that, after entering the systemic circulation, is able to be absorbed and reach the site of action. It is an important measure of the degree to which nutrients are utilized in the body [110]. Bioavailability is affected by many factors, including the chemical properties of nutrients, food intake, and the physiological state of the individual [110]. Bioavailability is one of the key parameters in the study of metabolic kinetics. It is closely related to the absorption, distribution, metabolism, and excretion of nutrients, which together affect the efficacy and safety of nutrients.

### 5.1. Absorption and Biotransformation

Anthocyanins are rapidly absorbed in the human oral cavity and appear in the blood soon after ingestion, and their degradation process begins in the oral cavity. In the oral cavity, anthocyanins may bind to oral epithelial cells and salivary components and be exposed to multiple enzymatic activities, which may lead to early degradation of anthocyanin [111]. Saliva and oral microbiota have β-glucosidase activity, so it is possible that anthocyanins may be partially deglycosylated in the oral cavity. Mallery et al. found that procatechin, a product of the microbial degradation of cyanidin-3-o-glucoside, can be detected in saliva in healthy volunteers after the ingestion of blackberry cyanidin-3-o-glucoside, which is hydrolyzed to glycosidic forms by β-glycosidase produced by oral epithelial cells and bacteria [112]. However, due to the short residence time of food in the oral cavity, these processes have limited impact on the bioavailability of anthocyanins, so most anthocyanins may enter the stomach in their native form [113].

Release from the food matrix is the first step in the availability of anthocyanins for gastrointestinal absorption. Under the highly acidic conditions prevalent in the stomach, anthocyanins should preferentially exist in their flavylium cation form. Although they are relatively stable in this form, they may also interact with charged molecules such as proteins, ionic carbohydrates, for example, pectin, and other polyphenols to form stable complexes, which may affect absorption and metabolism [114,115,116]. The oral relative bioavailability of pelargonidin glycosides in strawberry beverages was reported to be reduced by approximately 50% when prepared in milk [117]. Ribnicky et al. investigated the intestinal bioavailability of blueberry anthocyanins under different conditions using a human upper gastrointestinal tract model. They found that a lipid-rich matrix affects the accessibility of anthocyanins, an effect that, depending on the structure of the anthocyanin, may either increase or decrease its accessibility. However, when combined with a protein-rich matrix, such as nonfat soybean flour, anthocyanins are protected from passing through the upper digestive tract, thereby facilitating their further delivery in the colon [118].

Once in the small intestine, anthocyanins may occur as a mixture of several structural forms, flavonoids, quinones, hemiketene, and chalcones, in proportions that are difficult to predict [119]; still, given the near-neutral pH, the quinone and/or hemiketo forms are likely to dominate [120]. The hemiketene forms are thought to be more susceptible to oxidative degradation than the flavonium cations, which may lead to their breakdown to produce smaller phenolic products such as phenolic acids.

### 5.2. Distribution

Anthocyanins are mainly distributed in the digestive organs and can also be widely distributed in the tissues and blood of rats, and can cross the BBB to enter the brain. Anthocyanins are quite water-soluble and can be detected in the brain as early as 10 min after oral administration into the stomach. It has been proposed that bilirubin translocase plays a role in transporting flavonoids in the stomach, and studies have shown that this transporter can interact with anthocyanins [121]. The absorption mechanism of anthocyanins in the central nervous system may be similar to that in the stomach, and there are also transporters composed of biliary transport enzymes in the endothelial cells that constitute the BBB [41]. Anthocyanins can accumulate in the cerebral cortex, cerebellum, hippocampus, and striatum of rats at concentrations up to 0.21 nmol/g [122]. Shimazu et al. found that the BBB permeability of different types of anthocyanins was different through biological experiments, and some anthocyanins had better BBB permeability [123]. Meanwhile, in vitro studies showed that anthocyanins could accumulate in brain endothelial cells and cross the BBB within 1 h [124].

### 5.3. Gastrointestinal Metabolism

Metabolic transformation of anthocyanins involves the synergistic action of the host enzyme system and the gut microbiota. After entering the circulation, anthocyanins are taken up and metabolized by liver cells, where they undergo phase II metabolic reactions (e.g., glucuronidation, sulfation, or methylation) to generate more-polar metabolites (e.g., cyanidin glucuronide conjugates). These reactions are mainly catalyzed by uridine diphosphate glucuronosyltransferase and sulfotransferase to enhance water solubility and facilitate excretion [125].

The relevance of the colon as a site of anthocyanin catabolism and metabolite absorption was confirmed by Mueller et al., who compared anthocyanin bioavailability in healthy subjects with that of subjects with ileostomy and found that subjects with intact intestines had significantly higher levels of anthocyanins and degradants in plasma and urine [126]. A large number of microorganisms are present in the human colon, with a wide variety. These microbial groups possess a wide range of enzymatic activities and are capable of extensive catabolism of anthocyanins into a variety of simpler phenolic compounds through reactions such as deglycosylation, ring cleavage, decarboxylation, demethylation, and dihydroxylation. Normally, the gut microbiota participates in anthocyanin metabolism through the activities of enzymes such as β-glucosidase, β-glucuronidase, α-galactosidase, and α-rhamnosidase, leading to the cleavage of the glycosidic bond. After deglycosylation, the C ring of the glycosidic ligand is cleaved, resulting in the formation of simple phenols, such as gallic acid, protocatechuic acid, syringic acid, p-coumaric acid, vanillic acid, cinnamic acid, phenylpropionic acid, 2, 4, 6-trihydroxybenzaldehyde, etc. [127,128,129]. For example, De Ferrars et al., in an interventional study of postmenopausal women consuming anthocyanin-rich berry (Elderberry) extract, found 28 anthocyanin metabolites in urine and 21 anthocyanin metabolites in plasma (including vanillic acid, protocatechin, and sulfate of benzoic acid) [130].

In summary, the low bioavailability of anthocyanins is primarily attributed to the following reasons: Anthocyanins may be adsorbed by carbohydrates, proteins, or fibers in complex food matrices, limiting their release. Gastric acid and digestive enzymes can degrade anthocyanins, reducing their stability. The absorption rate of anthocyanins in the intestine is low, and they are easily metabolized by gut microbiota into other compounds. Factors such as age, health status, and gut microbiota composition also affect absorption efficiency. The bioavailability of anthocyanins is generally low, ranging from approximately 0.1% to 1.5%, and in rare cases, it may approach 5% [131].

## 6. Strategies to Enhance the Bioavailability and Stability of Anthocyanins

**Figure 3:**
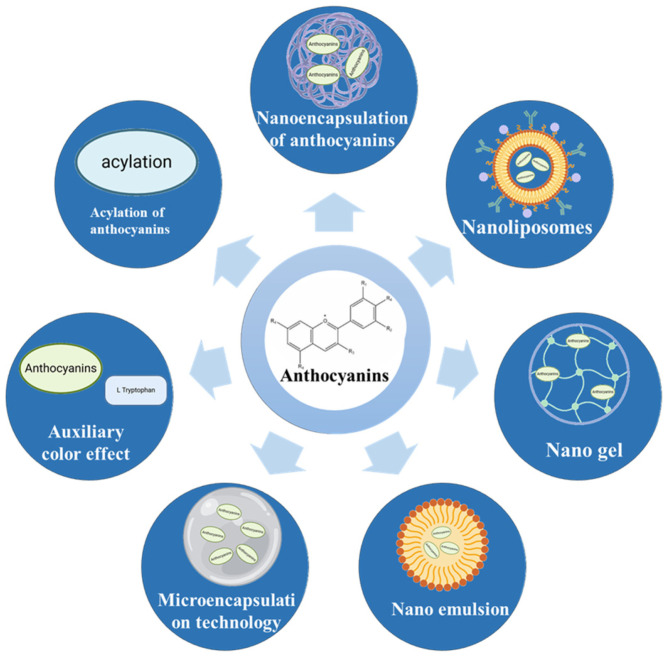
Strategies to enhance the bioavailability and stability of anthocyanins.

Anthocyanins, as potent natural polyphenolic compounds, have attracted much attention for their potential role in the prevention of central nervous system-related diseases. However, due to their sensitivity to certain environmental and gastrointestinal conditions, anthocyanins often exhibit low chemical stability and a short half-life as well as relatively low bioavailability, which limits their health benefits. To improve the stability and bioavailability of anthocyanins, effective technical improvements are essential. At present, there are two main methods to modify the structure of anthocyanins (Figure 3). The first method is to modify the structure and functional groups of anthocyanins to acylate hydroxyl groups to reduce water solubility and enhance stability. The second method is to form functional complexes with other materials or encapsulate anthocyanins into a carrier matrix, which can effectively protect anthocyanins from external adverse conditions, enhance their stability, and effectively improve their biological functions. Researchers are exploring a variety of approaches, including microencapsulation, encapsulation, and other techniques.

### 6.1. Glycosylation and Acylation of Anthocyanins

The clinical application of anthocyanins has been severely limited by their low blood–brain barrier (BBB) penetration efficiency. Glycosylation and acylation balance hydrophilicity and lipophilicity, enabling anthocyanins to penetrate the blood–brain barrier (BBB) and directly act on the central nervous system (e.g., inhibiting β-amyloid (Aβ) aggregation) [132]. Glycosyl groups are usually attached to position C3 or C5 via O-glycosidic bonds (e.g., cyanidin-3-O-glucoside). The membrane of 1-palmitoyl-2-oleoyl-sn-glycero-3-phosphocholine was used as a model to measure the interaction of anthocyanins with the membrane via electrophysiological techniques. It was found that glycosylation increased the water solubility of anthocyanins, thereby reducing their affinity for membrane lipids, but they were still able to affect the fluidity of the membrane [133].

In vitro experiments, including enzymatic acylation reactions and thermal and photostability tests, as well as antioxidant capacity assessments, have demonstrated that acylation can significantly enhance the stability and antioxidant capacity of anthocyanins [134]. Anthocyanin acylation refers to the acylation of the sugar group of anthocyanins with aromatic or aliphatic substituents, and is usually the final step in anthocyanin biosynthesis [135]. Acylation reduces the polarity of anthocyanins, alters their molecular size, and produces a steric effect that reduces the sensitivity of anthocyanins to nucleophilic attack, which in turn increases the structural and chemical stability of anthocyanins in vitro and in vivo [136]. Substituents for anthocyanin acylation fall into two major groups: aromatic acyl and fatty acyl. Aromatic acyl substituents are generally hydroxycinnamyl groups, such as p-coumaryl, coffeyl, ferrityl, and cineryl; fatty acyl substituents are mainly malonyl, acetyl, succinyl, and glycolyl, among which malonyl is the most widely distributed [135]. The acylation site, acyl type, and acyl number have a combined effect on anthocyanin synthesis. For example, unacylated anthocyanins will decolorize rapidly in neutral or weakly acidic aqueous solutions, but acylated anthocyanins will be more stable in aqueous solutions through intramolecular co-color and steric effects, protecting xanthine chromophors from nucleophilic attack of water [137]. The stability of polyacylated anthocyanins is higher than that of monoacylated anthocyanins. Under the same cell conditions, the polyacylation reaction with multiple aromatic substituents can improve the color stability of anthocyanins and make them more blue [137,138]. Substances that can acylate anthocyanins are as follows: caffeic acid, coumaric acid, ferulic acid, p-hydroxybenzoic acid, and other aromatic acids and fatty acids. Although no direct studies have been found on the application of anthocyanin acylation in central nervous system diseases, acylation modification is commonly used to improve the stability and bioavailability of bioactive molecules. Therefore, the acylation of anthocyanins may further enhance their protective effects in the central nervous system and may become a research direction in the future.

### 6.2. Copigmentation Effect

The copigmentation effect of amino acids is primarily achieved through non-covalent interactions such as hydrogen bonding with hydroxyl groups in anthocyanin molecules, π-π stacking, and van der Waals forces [139]. These interactions can inhibit the degradation pathways of anthocyanin molecules, thereby improving their stability under different environmental conditions. For example, it has been found that amino acids such as L-tryptophan, L-aspartic acid, and L-proline can significantly improve the stability of anthocyanins, and L-tryptophan, in particular, can significantly prolong the half-life of anthocyanins through hydrogen bonding interactions with anthocyanins [139]. In addition, it has been shown that there are differences in the stabilizing effects of different amino acids on anthocyanins. For example, L-proline and L-aspartic acid were able to significantly enhance the stability of anthocyanins in mulberry under conditions of light or ascorbic acid addition [140]. Furthermore, it was found that small peptides and amino acids produced by proteolysis could bind to anthocyanins through ionization or hydrophobic groups, thereby enhancing their stability. For example, collagen hydrolysates significantly extended the half-life of anthocyanins from 40.7 h to 251.1 h by forming non-covalent complexes with anthocyanins [139]. The copigmentation effect of amino acids not only improves the stability of anthocyanins, but also enhances their antioxidant activity. It has been found that amino acid residues can reduce oxidative damage by scavenging free radicals through hydrogen bonding and hydrophobic interactions with them. Peptides containing aromatic amino acids, such as tyrosine and tryptophan, exhibit strong free radical scavenging, which is attributed to the ability of their phenolic hydroxyl and indole groups to directly act as hydrogen donors to trap free radicals. In addition, this auxiliary color effect of amino acids may also make anthocyanins more prone to exert antioxidant effects by changing their molecular conformation. For example, peptides containing hydrophobic and aromatic amino acids were able to significantly enhance antioxidant capacity, which was mainly attributed to their synergistic effects [8].

The combination of amino acids with anthocyanins can improve the color performance of anthocyanins, making them more promising in food applications. Future studies could further explore the interaction mechanism between amino acids and anthocyanins to optimize the stability and bioavailability of anthocyanins.

### 6.3. Nanoencapsulation of Anthocyanins

Nanodrug delivery systems play an important role in the treatment of central nervous system diseases. Through surface modification (e.g., polyethylene glycol, specific targeting groups), nanocarriers can effectively penetrate the BBB and improve the targeting of drugs. Nanodrug delivery systems can enhance the efficacy of drugs through a variety of mechanisms, including improving drug bioavailability, reducing drug degradation, and enhancing drug accumulation at the lesion site [141].

Nanoencapsulation technology can effectively improve the stability and bioavailability of anthocyanins by combining them with biomaterials. Anthocyanins extracted from blackberries were nanoencapsulated using citrus pectin and lysozyme. This method resulted in nanostructures with a size of 190 nm, a zeta potential of –30 mV, and a spherical and homogeneous morphology. Nanoencapsulation not only enhanced the stability of anthocyanins but also increased their bioavailability and tissue delivery. In vivo studies showed that nanoencapsulated anthocyanins were absorbed more efficiently and distributed to various tissues such as the blood, spleen, bladder, pancreas, and bone, unlike unencapsulated anthocyanins which were mainly found in the kidneys and bladder [142]. This technology has shown great potential in the prevention and treatment of central nervous system diseases. Blueberries are rich in anthocyanins. Herrera-Balandrano et al. used ferritin nanocarriers or a combination of FR and sodium alginate to embed blueberry anthocyanin extract and anthocyanin standards (cyanidin and pelargonidin glycosides) [143]. Blueberry anthocyanin extract and anthocyanin standards were evaluated for stability and absorption in simulated gastrointestinal conditions and Caco-2 cell monolayers. The results showed that the use of ferritin nanocapsules was able to achieve sustained release of anthocyanins during simulated digestion. In particular, after 2 h in the intestinal stage, the concentration of anthocyanins in the blueberry anthocyanin extract embedded using ferritin nanocarriers was significantly higher (38.01 µg/mL, *p* < 0.05), compared with only 4.12 µg/mL in the untreated blueberry anthocyanin extract. In addition, the results of the Caco-2 cell monolayer assay showed that the anthocyanin uptake rate of ferritin-embedded cells was significantly higher than that of untreated cells.

Studies have shown that amphiphilic peptides can enhance the stability of anthocyanins, form protective structures through molecular self-assembly, and reduce the degradation of anthocyanins [144]. An amphiphilic peptide C6M1 consisting of 18 amino acids was used to encapsulate anthocyanins through a co-assembly mechanism. The size of the obtained peptide-encapsulated anthocyanin nanocomposites was less than 100 nm, and the encapsulation efficiency reached 77.06%. Studies have shown that the co-assembly of anthocyanins leads to a conformational change in the C6M1 peptide from a β-helix structure to a β-fold structure, which leads to the efficient encapsulation of anthocyanins. The intermolecular interaction between the C6M1 peptide and anthocyanins was further clarified by fluorescence quenching experiments. The C6M1 peptide significantly enhanced the tolerance of anthocyanins to pH change, metal ions, and a high-temperature environment, while retaining the inherent function of anthocyanins in scavenging free radicals. Moreover, the binding of anthocyanin-3-O-glucoside to silk fibroin significantly enhanced its tolerance to an alkaline environment, and the retention of anthocyanin-3-O-glucoside in different concentrations of Cu^2+^ solution was also greatly improved. At the same time, this binding resulted in a significant increase in the heat resistance of anthocyanin-3-O-glucoside at 80 °C, with a 2.5-fold increase in the average half-life compared with the unbound anthocyanin-3-O-glucoside. Notably, silk fibroin did not affect the antioxidant activity of anthocyanin-3-O-glucoside and successfully maintained its original functional properties [145,146].

### 6.4. Nanoliposomes

Liposomes are microvesicles with a cell-like structure, which are composed of an amphiphilic lipid wall material. Owing to their excellent biocompatibility and unique capacity to encapsulate both hydrophilic and hydrophobic substances, liposomes have emerged as a prominent research focus in the fields of food science and pharmaceutical development. 

Several authors have prepared nanoliposomes using anthocyanin standards and demonstrated their advantages in the treatment of diabetes and cataracts. Gharib et al. synthesized nanoliposomes containing chlorinated anthocyanin (Cy-NL) and chlorinated delphinidin (Dp-NL) by combining a dried lipid layer composed of soybean lecithin and cholesterol (molar ratio 6:1) with 150 mg/mL anthocyanin or chlorinated delphinidin [147]. Their in vitro study showed that 100 mg/mL Cy-NL and DP-NL could reduce the glycation rate of albumin by 85.4% and 91.5%, respectively, which was significantly better than that of Cy and Dp standards, which was reduced by 54.0% and 69.5%, respectively. In addition, it was found that 100 mg/kg nanoliposomes or standards administered intravenously daily for eight weeks to streptozotocin (50 mg/kg)-induced diabetic mice significantly reduced total cholesterol, glycated albumin, and glycated hemoglobin A1c, while increasing glycogen levels. Nanoliposomes were more effective than the standard in improving these parameters. In a gamma scintigraphy study utilizing a rabbit eye model, Zhang et al. demonstrated that N-trimethyl chitosan-coated cyanidin-3-glucoside nanoliposomes (TMC-Cy3G-NL), prepared via reverse-phase evaporation using lecithin and cholesterol, significantly prolonged pre-corneal retention time and enhanced deep transport to the corneal epithelium [148]. This highlights the potential of TMC-Cy3G-NL as an effective ocular delivery system.

In addition, this study also found that TMC-Cy3G-NL could alleviate selenite-induced oxidative stress in rats. In another antioxidant study, TMC-Cy3G-NL was shown to enhance the activities of superoxide dismutase and catalase, as well as reverse the decrease in reduced glutathione activity induced by sodium selenite, compared with uncoated cyanidin-3-glucoside. It is more effective in preventing oxidative stress and lipid peroxidation in the eye lens of SD rats.

In addition to nanoliposomes based on anthocyanin standards, the preparation and application of nanoliposomes enriched in natural extracts of anthocyanins were also investigated. Hwang et al. prepared multilayered nanolipids from hibiscus flowers using lecithin and cholesterol [149]. The results showed that the incorporation of 20 mg/mL and 50 mg/mL anthocyanin extract into the nanoliposomes significantly enhanced the 2,2-Diphenyl-1-picrylhydrazyl radical scavenging activity from 11% to 64% for the former and from 12% to 76% for the latter. In addition, the same concentration of multilayered nanoliposomes inhibited melanin production in human A375 melanoma cells significantly better than anthocyanin extract alone. It has also been found that multilayered vesicles exhibit greater tolerance to the harsh environmental conditions in the stomach and intestine compared with single-layered liposomes.

To prevent anthocyanin degradation induced by ascorbic acid, Guldiken et al. prepared nanoliposomes (BENCE-NL) containing different concentrations of black carrot extract (BCE) rich in anthocyanins (0.1%, 0.2%, and 0.4%) and soybean lectins (1%, 2%, and 4%) [150]. After 24 h of storage with different concentrations of ascorbic acid (0.01%, 0.025%, 0.05% and 0.1%), the color and stability of BCE and BCE-NL were observed to decrease in a dose- and time-dependent manner. However, the degradation rate of anthocyanins in BCE-NL was significantly reduced. This may be because the bilayer of vesicles in the liposomes acts as a barrier, protecting anthocyanins from degradation by ascorbic acid. Lafai et al. also compared the application of different concentrations of soybean lectins (1%, 2%, and 3%) as well as different concentrations of extracts (500, 750, and 1000 ppm) in the preparation of nanoliposomes containing pistachio green shell extract (PHE-NL) and evaluated the stability of anthocyanins [151]. Pistachio green shell extract is a rich source of phenolic compounds, which contains 118.6 mg cyanidin-3-glucoside/g of anthocyanins. Both monolayer and spherical nanoliposomes were prepared by the thin-film water method. PHE-loaded nanoliposomes were able to increase the phase transition temperature and the oxidation initiation temperature, indicating that higher thermal stability and lipid oxidation stability were achieved by polyphenol loading. In addition, only minor changes in particle size, polydispersion index, and zeta potential were observed after two months of storage at 4 °C, while 75% of the encapsulated polyphenols were retained.

Nanoliposomes can enhance the interaction with the endothelial cells of the BBB by surface modification of targeted ligands, such as transferrin, insulin, Apolipoprotein E, etc., so as to improve the penetration of drugs. For example, transferrin-modified liposomes can significantly improve the BBB permeability of drugs, and the apparent permeability is several times higher than that of unmodified liposomes. Nanoliposomes have become a promising drug delivery system for the treatment of central nervous system diseases. Although the application of anthocyanin nanoliposomes in the prevention and treatment of central nervous system diseases has not been reported, anthocyanin nanoliposomes may have broad prospect in the treatment of central nervous system diseases by overcoming the pharmacokinetic defects of natural anthocyanins. Future research needs to integrate the multidisciplinary advantages of materials science, computational biology, and clinical medicine to promote the upgrading of this natural compound into a precision neuroprotective agent.

### 6.5. Nanogel

Compared with other gels, nanogels can easily penetrate various protective membranes in the human body, such as meninges. Embedding anthocyanins in nanogels can achieve drug delivery in special sites such as the brain, and can enhance the chemical stability of anthocyanins, intestinal absorption, and cellular antioxidant capacity [152]. Feng et al. developed composite nanogels for the delivery of cyanidin-3-glucoside by combining the Maillard reaction and thermal gelation. The cyanidin-3-glucoside-loaded nanogel is spherical with a diameter of about 185 nm and maintains structural stability over a wide range of pH and NaCl concentrations. The composite nanogel enhanced the chemical stability of cyanidin-3-glucoside in an accelerated degradation model and in a simulated gastrointestinal tract, and nano-cyanidin-3-glucoside was more effective than free cyanidin-3-glucoside in restoring cell viability and endogenous antioxidant enzyme activity [153]. Gellan gum is a biopolymer that can be used as a material to protect and carry bioactive compounds, and this polysaccharide is resistant to gastric pH conditions. Santos et al. used two different values of ionic strength, with the addition of calcium ions, to prepare a Gellan-cooled gel containing the anthocyanin extract of Jabuticaba [154]. Cold gels were found to improve the retention of anthocyanins and can be used as good carriers of anthocyanins; however, the mechanical properties of gels change with the addition of Jabuticaba anthocyanin extract, so matrix modification caused by bioactive additives should be considered. Xie et al. prepared a new nanocomposite-embedded hydrogel system by co-coloring blueberry anthocyanins and chondroitin sulfate and incorporating the hydrogel into caragenan [155]. The results showed that chondroitin sulfate could maintain the stacking structure of blueberry anthocyanins due to its high charge density.

Colorimetric volatile amine sensors for anthocyanins have received much attention in recent years for monitoring the freshness of meat and fish. However, most sensors suffer from anthocyanin loss in humid packaging environments, severely limiting their indicative ability. Zhai et al. developed a hydrogel–oil bigel to prevent anthocyanin loss [152]. The inner hydrogel was composed of water, agar, and purple sweet potato anthocyanins, and the outer oil gel was composed of sunflower oil, beeswax, and Glyceride Monooleate. The double gel was extruded onto the polyvinylidene fluoride (PVDF) film by a 3D printing method to form a composite film. The results showed that the anthocyanins in the PVDF–double gel composite film exhibited good stability in water and showed strong anti-loss properties. Multiple studies have found that various intelligent films prepared using zein, starch, polyvinyl alcohol, sodium alginate, chitosan, ethylcellulose, castor oil, methylcellulose matrices, and different types of anthocyanins significantly enhance the color stability of anthocyanins compared to pure anthocyanins alone. These films hold increasing potential application value in the field of intelligent food packaging [156,157,158,159,160,161,162,163,164,165,166]. By adding carrot extract to the lower agar film and titanium dioxide to the upper agar film, Yang et al. developed a bioactive agar bilayer, which significantly improved the barrier and mechanical properties of the bilayer, effectively maintaining the total anthocyanin content and enhancing the stability of anthocyanin [167]. Liu et al. developed a novel bilayer colorimetric film by combining polycaprolactone with anthocyanins extracted from *Clitoria ternatea* Linn using electrospinning. This innovative design enhances the stability of anthocyanins, allowing the film to withstand harsh environmental conditions that typically degrade anthocyanin stability [168].

### 6.6. Nanoemulsions

Nanoemulsions are kinetically stable but thermodynamically unstable colloidal systems formed by mixing oil, emulsifier, and water [169]. The reasons for using nanoemulsions to improve the stability of anthocyanins include physical protection, chemical stability, environmental regulation, and improved bioavailability [170]. Physically, the small size and multilayer structure of nanoemulsions reduces the exposure of anthocyanins to the external environment and prevents oxidation and degradation. Chemically, surfactants and antioxidants can form stabilizing bonds with anthocyanins and enhance their chemical stability. In terms of environmental regulation, nanoemulsions can maintain a stable acidic and temperature environment, reducing temperature variation and the catalytic degradation of metal ions. In addition, nanoemulsions can also improve the bioavailability of anthocyanins and prolong their action time in vivo through sustained release, while reducing the direct effect of light on them [8,171]. These mechanisms work together to significantly improve the stability and activity of anthocyanins.

Pratiwi et al. prepared nanoemulsions of anthocyanin-rich mangosteen peel extract with the aim of developing a self-emulsified drug delivery system (SEDDS) by a simplex lattice design approach. The optimal SEDDS, consisting of coconut oil, Tween 80, and polyethylene glycol 400 at a ratio of 1:6.95:2.05, was obtained by adding ethyl acetate extract [172]. This SEDDS showed a higher diffusivity (97%) within 8 h in an in vitro Franz diffusion permeation model compared to the unloaded SEDDS (19%), indicating that the SEDDS formulation containing mangosteen peel extract could increase the penetration of the major component α-mangosin through the cuticle. Chen et al. encapsulated blueberry anthocyanins (BAE) into a microemulsion system (BAE-ME). They developed a pseudo-ternary phase diagram using isopropyl myristate, Tween 80/Span 80, and ethanol as the oil, surfactant, and co-surfactant, respectively, to create a stable water-in-oil (W/O) BAE-ME system. It has been shown that anthocyanins incorporated into ME for encapsulation have higher retention than free anthocyanins at 4 °C and 25 °C. Furthermore, BAE-ME remained stable at an ionic strength ≤ 1.0 mol/L NaCl and in an up to 9% sugar solution (glucose–sucrose combination) [173]. Rabelo et al. developed a water-in-oil (W/O) nanoemulsion to encapsulate anthocyanins from acai berries [174]. They combined a medium-chain triglyceride oil phase containing an emulsification agent with acai berry aqueous solutions at varying weight fractions (10% to 30%). After storing the samples at 4 °C in the dark for 30 days, the nanoemulsion containing 2% 2% AE encapsulated in a 30 wt% W/O nanoemulsion had an estimated half-life of 385 days.

### 6.7. Microencapsulation Technology

Microcapsule embedding has proved to be a practical technique for stabilizing and delivering anthocyanins. Kanokpanont et al. used an in vitro gelation process to prepare calcium alginate/chitosan composite microspheres embedded with mulberry leaf anthocyanin extracts. The results showed that the calcium alginate microspheres prepared in 0.05% chitosan solution were suitable for the extract of mulberry leaf anthocyanins, and the encapsulation efficiency was high. Each 1 g of calcium alginate microspheres could encapsulate 2.7 mg of anthocyanins, and about 60% of anthocyanins could be retained in gastric juice for 24 h [175].

## 7. Conclusions and Future Research Directions

In recent years, anthocyanins have shown unique neuroprotective potential in the field of the prevention and treatment of central nervous system diseases. Anthocyanins provide new ideas for intervention in neurodegenerative diseases and various nerve injuries through the molecular mechanism of multi-target regulation of oxidative stress, inflammatory response, and apoptosis. Existing studies have confirmed that anthocyanins have significant protective effects in PD, cerebral ischemia–reperfusion injury, chemotherapy-related neurotoxicity, and other disease models, especially in the regulation of Nrf2/ARE, NF-κB, PI3K/AKT, NLRP3/caspase-1/IL-1β, and other key signaling pathways. However, its natural form faces bottlenecks such as poor chemical stability and low bioavailability, which seriously restrict clinical application and transformation.

Future research should focus on three dimensions. First, directed protection and targeted release of anthocyanins in the gastrointestinal environment can be achieved through the innovative design of nano-delivery systems, such as the development of metal–organic frameworks (MOFs) or liposome composite carriers with pH-responsive properties. Second, an in-depth understanding of the interaction mechanism between anthocyanin metabolites and gut microbiota may reveal the neuroprotective effects of new active metabolites and provide a theoretical basis for the development of prodrug formulations. Of interest, recent studies have suggested that the interaction between anthocyanins and epigenetic regulatory elements (such as histone deacetylases and DNA methyltransferases) may open a new dimension of neuroprotection, which provides a direction for the development of epigenetically antioxidant synergistic therapies. In the future, it will be possible to establish an artificial intelligence-based molecular simulation platform to enhance the BBB penetration of anthocyanins through structural modification while retaining their pharmacophores. With the deep integration of precision delivery technology and multi-omics analysis technology, anthocyanins are expected to break through the existing limitations and become the core components of the next generation of intelligent neuroprotective agents.

## Data Availability

No new data were created or analyzed in this study. Data sharing is not applicable to this article.

## Abbreviations

The following abbreviations are used in this manuscript:

DALY	Global disability-adjusted life years
AD	Alzheimer’s disease
PD	Parkinson’s disease
ALS	Amyotrophic lateral sclerosis
BBB	Blood–brain barrier
Nrf2	Nuclear factor erythroid 2-related factor 2
MPTP	1-methyl-4-phenyl-1, 2, 3, 6-tetrahydropyridine
ROS	Reactive oxygen species
MPP+	1-methyl-4-phenylpyridinium
APP/PS1	APPswe/PSEN1dE9
PI3K/	Phosphoinositide 3-kinase
Akt	Protein kinase B
IL-1β	Interleukin-1β
IL-6	Interleukin-6
TNF-α	Tumor necrosis factor-alpha
NF-κB	Nuclear factor kappa-light-chain-enhancer of activated B cells
MAPK	Mitogen-activated protein kinase
CREB	Monophosphate response element-binding protein
hSOD1G93A	G93A mutant human superoxide dismutase 1
NLRP3	NOD-like receptor family, pyrin domain containing 3
PVDF	Polyvinylidene fluoride
SEDDS	Self-emulsified drug delivery system
HDACs	Histone deacetylases
DNMTs	DNA methyltransferases
